# Perioperative Red Blood Cell Transfusion and Long-Term Mortality in Coronary Artery Bypass Grafting: On-Pump and Off-Pump Analysis

**DOI:** 10.3390/jcm14082662

**Published:** 2025-04-13

**Authors:** Seung Hyung Lee, Ji Eon Kim, Jun Ho Lee, Jae Seung Jung, Ho Sung Son, Hee-Jung Kim

**Affiliations:** Department of Thoracic and Cardiovascular Surgery, Korea University Anam Hospital, College of Medicine, Korea University, Seoul 02841, Republic of Korea; toohardlogname@gmail.com (S.H.L.); jieonkim82@gmail.com (J.E.K.); ecmo1984@gmail.com (J.H.L.); heartistcs@korea.ac.kr (J.S.J.)

**Keywords:** coronary artery bypass grafting, red blood cell, transfusion, mortality

## Abstract

**Background/Objectives**: The impact of different coronary artery bypass grafting (CABG) strategies, particularly on-pump versus off-pump techniques, on red blood cell (RBC) transfusions and their associated outcomes has not been fully investigated. This study aims to evaluate the association between RBC transfusion and survival in CABG patients, focusing on-pump strategy. **Methods**: Data from CABG patients were retrieved from the National Health Insurance Service database (2003 to 2019). Perioperative RBC transfusions were classified into three groups: no transfusion, RBC 1, and RBC ≥ 2 units. The primary endpoint was all-cause mortality rate. Subgroup analysis assessed the impact of RBC transfusion on mortality across the conventional on-pump (CCAB) and off-pump (OPCAB) groups. **Results**: Among the 6150 participants who underwent CABG, 2028 underwent CCAB and 4122 underwent OPCAB. The mean age was 66.2 ± 9.7 years, with a mean follow-up of 2.9 (2.53–3.35) years. Multivariable analysis showed a significant association between transfusion of ≥2 RBC units and increased mortality risk (HR 2.34 [1.65–3.32], *p* < 0.001). Subgroup analysis showed a similar trend in both CCAB and OPCAB groups (*p* for interaction = 0.2). Transfusion of ≥2 units significantly increased mortality in OPCAB (HR 2.28 [1.55–3.37], *p* < 0.001) but not in CCAB (HR 2.96 [0.97–9.06], *p* = 0.057). OPCAB and surgery at large volume center was associated with a reduced risk of RBC transfusion (*p* < 0.01). **Conclusions**: Increased RBC transfusion is associated with higher long-term mortality in patients undergoing CABG. Based on a large cohort predominantly consisting of OPCAB patients, OPCAB is associated with decreased RBC transfusion requirements.

## 1. Introduction

Coronary artery bypass grafting (CABG) is commonly performed during cardiac surgery to treat ischemic heart disease and acute coronary syndrome [1]. Patients undergoing cardiac surgery, including CABG, occasionally need transfusion [2,3]. Transfusion is aimed at enhancing oxygen delivery to tissues by increasing hemoglobin levels [4]. However, perioperative transfusion is associated with worse outcomes [5,6,7,8]. These outcomes include increased morbidity in critically ill patients, as well as an increased risk of renal failure and respiratory, cardiac, and neurologic complications [9,10,11].

Despite efforts to reduce their incidence in patients undergoing surgery, transfusions commonly occur during CABG. Conventional on-pump coronary artery bypass grafting (CCAB) utilizes cardiopulmonary bypass (CPB) to temporarily take over heart and lung function during surgery. In contrast, off-pump coronary artery bypass grafting (OPCAB) is performed on a beating heart without the use of CPB, potentially reducing complications related to extracorporeal circulation [8,12]. The initiation of CPB necessitates a higher level of anticoagulation, significant manipulation of major vessels (such as the aorta and vena cava), and depletion of coagulation factors, all of which may contribute to an increased tendency for bleeding. However, the impact of CABG strategy, whether conventional on-pump or off-pump, on red blood cell (RBC) transfusion and clinical outcomes has not been thoroughly investigated.

Therefore, this study aimed to investigate the effects of RBC transfusion during CABG, specifically utilizing the OPCAB-dominant approach, on long-term mortality using a large administrative population-based database.

## 2. Materials and Methods

### 2.1. Study Participants

The data were obtained from the National Health Insurance Service (NHIS). Individuals possessing Korean nationality residing in Korea were mandated to participate in the NHIS. Consequently, the NHIS patient database epitomizes the medical practices prevalent in Korea and constitutes the largest repository of claims data in the country. The claims data utilized for this investigation encompassed patients’ demographic details, diagnoses, procedures, and prescription records pertaining to both inpatient and outpatient services. Between 2002 and 2019 (available in the NHIS), 76,233 patients underwent their first cardiac surgeries. Among these, 12,290 patients underwent isolated CABG. To obtain laboratory data collected before surgery, patients with health screening data within two years before the index surgery were included (*n* = 6140). The Institutional Review Board approved this study (IRB no. 2022AN0011), and the requirement for informed consent was waived.

### 2.2. Surgical Techniques

All the patients included in this study underwent isolated CABG. CPB strategies were classified into two groups for subgroup analysis: CCAB and OPCAB.

### 2.3. Variables in Analysis

Perioperative red blood cell (RBC) transfusion is operationally defined as the administration of transfused blood occurring both prior to and subsequent to the indexed coronary artery bypass grafting (CABG) procedure. Regarding the RBC transfusion categorization, the volume of transfused red blood cells during the perioperative period was stratified for analytical purposes into the following groups: 0 (no transfusion), 1 (RBC 1), and ≥2 (RBC ≥ 2) units. Anemia is clinically defined as a hemoglobin concentration of less than 13 g/dL in male patients or less than 12 g/dL in female patients.

### 2.4. Outcomes of Interest

The primary outcome of interest was the overall all-cause mortality during the postoperative follow-up period. Secondary study outcomes included identifying the risk factors associated with RBC transfusion in patients undergoing CABG.

### 2.5. Statistical Analysis

The risk of incident events based on the number of RBC transfusions was analyzed using the Kaplan–Meier method and log-rank test. Cox proportional hazards models were applied to evaluate the relationship between RBC transfusions and mortality, employing a backward elimination method for variable selection. The final model retained variables with a significance level of *p* > 0.2. In the multivariable analysis, the covariates that were adjusted for encompassed age, sex, estimated glomerular filtration rate (eGFR), body mass index (BMI), hypertension, diabetes mellitus, dyslipidemia, ischemic stroke, congestive heart failure, liver cirrhosis, a history of malignancy, atrial fibrillation, myocardial infarction, peripheral artery disease, the utilization of antiplatelet agents, admission via the emergency department, the application of extracorporeal membrane oxygenation (ECMO), surgical procedures conducted at high-volume centers (defined as centers performing more than 50% of surgical interventions; median cases: 324 [IQR, 290.5–474.5] for high-volume centers vs. 26 [IQR, 7–46] for low-volume centers), the volume of red blood cell (RBC) transfusions, and the comparative analysis between coronary artery bypass grafting (CCAB) and off-pump coronary artery bypass grafting (OPCAB). Individuals with incomplete datasets were excluded from the analysis. In order to evaluate the proportional hazard assumption, an interaction term involving RBC transfusion and temporal factors was incorporated into the Cox regression model. The findings indicated that the interaction term did not achieve statistical significance (*p* = 0.953), suggesting that the influence of RBC transfusion on survival remained consistent over time, thereby fulfilling the criteria for the assumption of proportional hazards.

A subgroup analysis utilizing various pump strategies was executed through Cox proportional hazards models to investigate the correlation between mortality and red blood cell transfusions within both the CCAB and OPCAB cohorts. The variables included in the multivariate analysis were consistent with those described above. The *p*-value for the interaction was calculated to assess the differential impact of RBC transfusions on mortality between the CCAB and OPCAB groups.

The transfusion risk was evaluated using a logistic regression model. In the multivariate model, the variables included in the analysis were consistent with those described above.

All statistical analyses were performed with SAS version 9.3 (SAS Institute, Cary, NC, USA) and R software version 4.0.5 (R Foundation, Vienna, Austria). Statistical significance was defined as a two-tailed *p* < 0.05.

## 3. Results

The study population included 6150 patients. The patient demographics are shown in Table 1. CCAB and OPCAB were performed in 2028 and 4122 patients, respectively. The mean follow-up duration was 2.9 (2.53–3.35) years. A total of 927 (15.07%) patients had anemia preoperatively and 4809 (78.2%) patients underwent RBC transfusions. Compared to CCAB, OPCAB patients had a significantly lower proportion of congestive heart failure, atrial fibrillation, myocardial infarction, and emergency admission. The mean number of transfused RBC units in the study population was 1.64 (±1.71). Although the proportion of patients with preoperative anemia did not differ according to CABG strategy, patients who underwent OPCAB received fewer transfusions than those who underwent CCAB (69.77% vs. 95.32%) (Table 1). The mean RBC transfusion volume during CCAB was also significantly higher than that during OPCAB (2.49 [±2.08] vs. 1.22 [±1.30]).

### 3.1. RBC Transfusion and Overall Mortality

**Figure 1:**
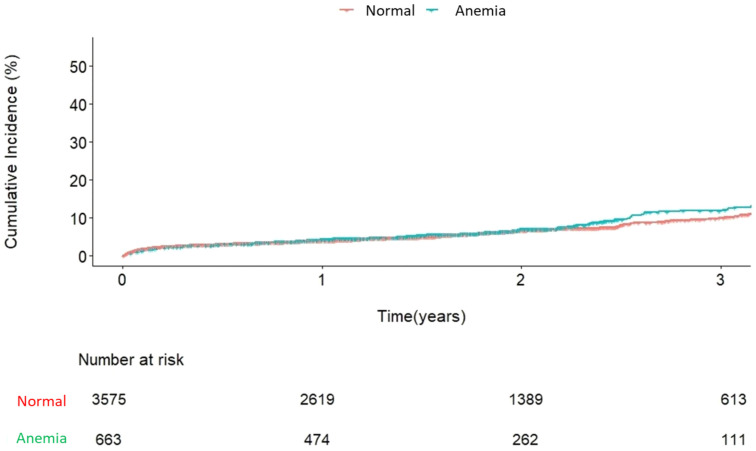
Incidence of mortality according to preoperative anemia.

**Figure 2:**
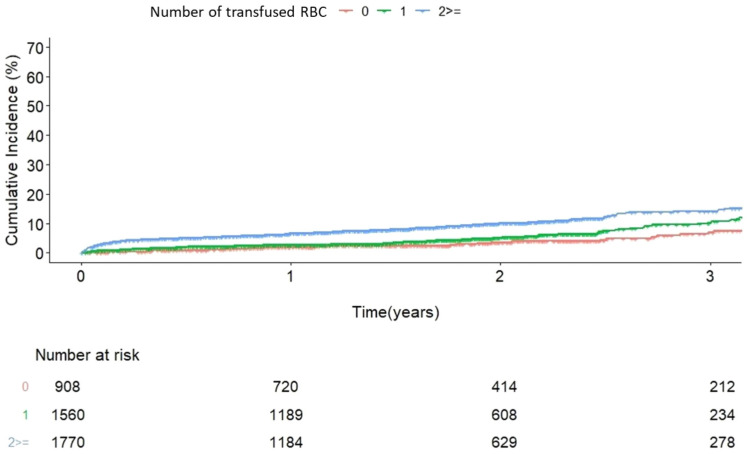
Incidence of mortality according to the RBC transfusion volume. RBC, red blood cell.

During the follow-up period, the overall mortality rate was 16.0% (983 of 6150 patients). In univariate analysis, preoperative anemia and transfusion volume were associated with an increased risk of mortality. In the multivariable analysis, transfusion volume remained an independent risk factor for mortality (RBC 1: hazard ratio [HR] 1.4 (0.97–2.01), *p* = 0.071; red blood cell [RBC] ≥ 2: HR 2.34 (1.65–3.32), *p* < 0.0001), while preoperative anemia did not show a statistically significant association (*p* = 0.8) (Table 2 and Figure 1 and Figure 2).

In subgroup analysis, the mortality rates for OPCAB and CCAB were 13.31% and 21.40%, respectively. A similar association was observed in both CCAB and OPCAB groups (*p* for interaction = 0.2). Transfusion of ≥2 units (RBC ≥ 2) was associated with an increased risk of mortality; however, this association was not statistically significant in the CCAB group (CCAB group: HR 2.96 (0.97–9.06), *p* = 0.057); (OPCAB group: HR 2.28 (1.55–3.37), *p* < 0.0001) (Table 3). All variables and statistical analyses of the subgroups are presented in Supplementary Tables S1 and S2.

### 3.2. Risk Factors for RBC Transfusion

In the multivariable analysis, factors associated with an increased prevalence of RBC transfusion included older age, reduced estimated glomerular filtration rate (eGFR), congestive heart failure, myocardial infarction, emergency admission, perioperative ECMO use, surgery performed at low-volume centers, preoperative anemia, and the conventional coronary artery bypass grafting (CCAB) technique compared to OPCAB (*p* < 0.05) (Table 4). These findings underscore the importance of hospital surgical volume, perioperative critical illness, and surgical technique in determining RBC transfusion rates.

## 4. Discussion

Based on our findings, RBC transfusion is associated with an increased risk of death. However, we did not observe any association between preoperative anemia and death. In the subgroup analysis, this relationship was observed in both the CCAB and OPCAB groups, but statistical significance was only observed in the OPCAB group. Additionally, the prevalence of RBC transfusions was higher in the CCAB group than in the OPCAB group.

RBC transfusions can increase oxygen delivery to tissues, which is helpful in myocardial ischemia [4]. Thus, RBC transfusion could be effective treatment, especially in surgery with hemorrhage, such as cardiac surgery or hemorrhagic shock. However, it is also associated with increase morbidity and mortality [5,6,7,13,14]. Therefore, all efforts should be made to minimize exposure.

Patients undergoing cardiac surgery who receive packed RBC transfusions have worse outcomes, including ischemic stroke, renal injury, and mortality, than non-transfused patients, even after adjusting for a variety of risk factors [14]. The adverse effects of RBC transfusion on outcomes can be explained by systemic inflammation [15]. This is because stored RBCs can lead to increased red blood cell aggregation and diminished microvascular autoregulatory ability. In addition, pro-inflammatory cytokines and potentially toxic microparticles are observed [9,16,17].

Patients with anemia are prone to tissue hypoxia; consequently, physicians tend to manage this condition by administering RBC transfusions perioperatively. This study also found that preoperative anemia was associated with RBC transfusion [13,18]. Because of this finding, many studies have suggested that anemia and RBC transfusion can independently affect outcomes [18,19,20,21,22]. Conversely, other studies have suggested that preoperative anemia itself may not be an independent risk factor for worse outcomes [23,24]. The current study found that the impact of RBC transfusion on survival was more pronounced than that of preoperative anemia.

Regarding the CPB strategy during CABG, OPCAB is associated with fewer RBC transfusions than CCAB [25,26]. This tendency could be attributed to the differences in the CPB strategies and the invasiveness of the techniques. Many studies have reported that OPCAB yields better short-term outcomes compared with those of CCAB [12,27]. However, OPCAB poses some potential hazards to survival, including technical difficulties, instrument issues, and anastomosis strategies. Thus, long-term survival according to the CPB strategy has not yet been clarified [28,29,30]. In the current study, the mortality rate in the OPCAB group was lower than that in the CCAB group (13.31% vs. 21.4%, respectively). This result can be attributed to the preference for OPCAB in Korea and its association with high-performance medical centers. In addition, the multivariable analysis indicated that the relationship between the RBC transfusion volume and increased mortality was not significantly affected by the operative technique.

In terms of risk factors associated with RBC transfusion, OPCAB procedures, treatment at high-volume hospitals, and the absence of ECMO support were identified as significant protective factors. However, these relationships are nuanced. OPCAB procedures were predominantly conducted at high-volume centers, which typically employ advanced patient blood management protocols and have more experienced medical teams compared to lower-volume centers [31]. Furthermore, aside from the inherent demands of extracorporeal support (such as CPB or ECMO), targeted training and implementation of effective patient blood management practices could significantly reduce the necessity for RBC transfusions [32].

### Limitation

Our investigation is subject to numerous constraints. Initially, notwithstanding its classification as a substantial population-based study, it possesses a retrospective design. Furthermore, the transfusion methodologies and technical interventions aimed at minimizing hemorrhage exhibited considerable variability across the 104 medical institutions offering cardiothoracic surgical programs in Korea. Due to the large number of hospitals during long period involved, factors such as blood transfusion thresholds and technical details, including pump operation, bypass conduit choice, and specific blood management protocols, were not investigated or incorporated into the analysis. These factors are significant considerations that can influence transfusion practices and outcomes, highlighting the need for further studies.

Second, our study could not definitively establish causality between RBC transfusion and associated outcomes due to its retrospective design and the lack of hemoglobin data at the time of transfusion or details regarding bleeding events. Without information on hemoglobin levels maintained during transfusion, the precise impact of RBC transfusion on mortality remains uncertain.

Third, our study was susceptible to selection bias due to the preference for off-pump CABG, which is widely performed in Korea. Consequently, the demographics of the patients who underwent conventional on-pump CABG in our cohort were worse than those reported in other studies. To mitigate this limitation, we incorporated numerous variables into our analysis, although some limitations persist.

Another limitation of our study was the higher transfusion rate observed compared with the other study groups. This discrepancy may be attributed to the relatively recent development of blood transfusion regulations and recommendations in Korea, primarily driven by referral medical centers and government initiatives. As a result, there exists a possibility of bias arising from disparities in transfusion methodologies when contrasted with practices in other nations. Additional investigation is essential to rectify these constraints and to furnish more thorough understandings regarding the correlation between red blood cell transfusion and patient prognoses.

Groups were categorized based on transfusion volume to achieve balanced case numbers across three groups. Because the number of cases with no transfusion or more than three units was limited, the threshold for categorization was set at one unit of transfusion.

In conclusion, our study reinforces the notion that RBC transfusion volume is an independent risk factor for survival, regardless of preoperative anemia. Additionally, OPCAB and higher surgery volume were associated with reduced RBC transfusion rates compared to CCAB. Reducing RBC transfusions during CABG may improve patient survival.

## Figures and Tables

**Table 1:** Demographic characteristics of the study cohort.

Variable	Total Cohort (N = 6150)	Conventional CABG Group (N = 2028)	Off-pump CABG Group (N = 4122)	*p*-Value
Age (year, mean, SD)	66.20 (9.72)	66.36 (9.63)	66.11 (9.76)	0.341
Sex (Female population, %)	1385 (22.54)	507 (25.02)	878 (21.32)	0.001
BMI	24.76 (3.01)	24.74(3.05)	24.77 (2.99)	0.697
Hypertension	5269 (85.67)	1735 (85.55)	3534 (85.74)	0.847
Diabetes	4131 (67.17)	1371 (67.60)	2760 (66.96)	0.612
Dyslipidemia	5509 (89.58)	1772 (87.38)	3737 (90.66)	<0.0001
Ischemic stroke	1259 (20.47)	413 (20.36)	846 (20.52)	0.884
Congestive heart failure	1959 (31.85)	721 (35.55)	1238 (30.03)	<0.0001
Liver cirrhosis	148 (2.41)	55 (2.71)	93 (2.26)	0.273
Malignancy history	815 (13.25)	244 (12.03)	571 (13.85)	0.048
Atrial fibrillation	448 (7.28)	192 (9.47)	256 (6.21)	<0.0001
Myocardial infarction	1618 (26.31)	608 (29.98)	1010 (24.50)	<0.0001
Peripheral artery disease	1215 (19.76)	375 (18.49)	840 (20.38)	0.081
Anti-platelet agent	4285 (69.67)	1361 (67.11)	2924 (70.94)	0.0021
Emergency admission	1777 (28.89)	798 (39.35)	979 (23.75)	<0.0001
ECMO use	265 (4.31)	174 (8.58)	91 (2.21)	<0.0001
Surgery at high volume center	2830 (46.02)	484 (23.87)	2346 (56.91)	<0.0001
Hemoglobin				0.403
Normal	5223 (84.93)	1715 (84.57)	3508 (85.10)	
Mild anemia (men 11–12.9 g/dL; women 11–11.9 g/dL)	692 (11.25)	226 (11.14)	466 (11.31)	
Moderate and severe (men < 10.9 g/dL; women < 10.9 g/dL)	235 (3.82)	87 (4.29)	148 (3.59)	
eGFR (mL/min)				0.042
eGFR ≥ 90	1376 (26.23)	435 (25.89)	941 (26.40)	
eGFR < 90 and ≥60	2821 (53.78)	888 (52.86)	1933 (54.22)	
eGFR < 60 and ≥30	827 (15.77)	267 (15.89)	560 (15.71)	
eGFR < 30	221 (4.21)	90 (5.36)	131 (3.67)	
Mean transfusion unit (count, SD)	1.64 (1.71)	2.49 (2.08)	1.22 (1.30)	<0.0001
Patients by transfusion volume				<0.0001
No transfusion	1341 (21.80)	95 (4.68)	1246 (30.23)	
RBC 1 unit	2204 (35.84)	576 (28.40)	1628 (39.50)	
RBC ≥ 2 units	2605 (42.36)	1357 (66.91)	1248 (30.28)	

SD, standard deviation; CABG, coronary artery bypass grafting; eGFR, estimated glomerular filtration rate; BMI, body mass index; high volume center, index surgery performed at large cases center (performing 50% of total cases); RBC, red blood cell.

**Table 2:** Multivariable analysis for overall death in the total cohort.

Variable	Hazard Ratio (95% CI)	*p*-Value
Age (years)		
20–40	Reference	
60–80	2.83 (0.62–13.00)	0.181
80≤	5.55 (1.19–25.95)	0.03
Female sex	0.78 (0.61–0.98)	0.034
eGFR (mL/min)		
eGFR ≥ 90	Reference	
eGFR < 60 and ≥30	1.73 (1.28–2.34)	<0.0001
eGFR < 30	3.56 (2.43–5.23)	<0.0001
Congestive heart failure	1.57 (1.29–1.92)	<0.0001
Ischemic stroke	1.32 (1.08–1.63)	0.008
Malignancy history	1.56 (1.22–1.98)	<0.0001
Atrial fibrillation	1.57 (1.19–2.08)	0.002
Peripheral artery disease	1.40 (1.14–1.73)	0.002
Emergency admission	1.22 (1.00–1.50)	0.053
ECMO use	10.81 (7.98–14.64)	<0.0001
Surgery at high volume center	0.64 (0.52–0.79)	<0.0001
RBC transfusion		
Number of RBC, 0	Reference	
Number of RBC, 1	1.40 (0.97–2.01)	0.071
Number of RBC, 2≤	2.34 (1.65–3.32)	<0.0001

eGFR, estimated glomerular filtration rate; BMI, body mass index; ECMO, extracorporeal membrane oxygenation; RBC, red blood cell; OPCAB, off-pump coronary artery bypass grafting; surgery at high-volume centers (defined as centers performing more than 50% of surgery cases).

**Table 3:** Multivariable analysis of long-term mortality in subgroup (conventional coronary artery bypass grafting (CCABG) versus off-pump coronary artery bypass grafting (OPCAB).

	Conventional CABG		OPCAB	
	HR (95% CI)	*p*-Value	HR (95% CI)	*p*-Value
Anemia				
Normal	Reference		Reference	
Anemia	0.84 (0.47–1.51)	0.565	1.21 (0.76–1.92)	0.429
Transfusion				
No transfusion	Reference		Reference	
RBC 1 unit	1.72 (0.55–5.46)	0.354	1.34 (0.90–2.00)	0.152
RBC ≥ 2 units	2.96 (0.97–9.06)	0.057	2.28 (1.55–3.37)	<0.0001

RBC, red blood cell; CI, confidence interval; HR, hazard ratio.

**Table 4:** Multivariable analysis for the risk of RBC transfusion.

Variable	Odd Ratio (95% CI)	*p*-Value
Age (years)		
20–40	Reference	
40~60	2.23 (0.90–5.54)	0.083
60~80	3.02 (1.22–7.48)	0.017
80≤	7.75 (2.82–21.28)	<0.0001
Female sex	1.90 (1.50–2.42)	1.90 (1.50–2.42)
eGFR (mL/min)		
eGFR ≥ 90	Reference	
eGFR < 90 and ≥60	0.95 (0.79–1.14)	0.567
eGFR < 60 and ≥30	1.44 (1.07–1.93)	0.015
eGFR < 30	4.43 (1.72–11.40)	0.002
Anemia	4.34 (2.95–6.38)	<0.0001
BMI (kg/m^2^)		
<18.5	Reference	
18.5–23	0.59 (0.20–1.79)	0.355
23–25	0.49 (0.16–1.50)	0.211
25–30	0.39 (0.13–1.22)	0.105
30<	0.26 (0.08–0.90)	0.033
Hypertension	1.05 (0.82–1.34)	0.696
Diabetes	1.08 (0.89–1.31)	0.457
Dyslipidemia	0.91 (0.66–1.26)	0.587
Congestive heart failure	1.30 (1.07–1.57)	0.008
Ischemic stroke	0.87 (0.70–1.08)	0.214
Liver cirrhosis	1.05 (0.59–1.84)	0.875
Malignancy history	0.99 (0.78–1.27)	0.938
Atrial fibrillation	1.19 (0.83–1.70)	0.351
Myocardial infarction	0.76 (0.62–0.93)	0.007
Peripheral artery disease	1.08 (0.87–1.34)	0.480
Anti-platelet agent medication	0.98 (0.80–1.20)	0.844
Emergency admission	1.28 (1.05–1.57)	0.017
ECMO use	7.49 (2.71–20.72)	<0.0001
Surgery at high volume center	0.78 (0.66–0.93)	0.005
OPCAB	0.12 (0.09–0.15)	<0.0001

eGFR, estimated glomerular filtration rate; BMI, body mass index; ECMO, extracorporeal membrane oxygenation; RBC, red blood cell; OPCAB, off-pump coronary artery bypass grafting; surgery at high-volume centers (defined as centers performing more than 50% of surgery cases).

## Data Availability

Restrictions apply to the availability of these data. Data were obtained from Korea National Health Insurance Service and are available at https://nhiss.nhis.or.kr/bd/ay/bdaya001iv.do with the permission of Korea National Health Insurance Service (accessed on 30 December 2024).

## Supplementary Materials

The following supporting information can be downloaded at: https://www.mdpi.com/article/10.3390/jcm14082662/s1, Supplemental Table S1. Multivariable analysis for overall death in conventional on-pump coronary artery bypass grafting group; Supplemental Table S2. Multivariable analysis for overall death in off-pump coronary artery bypass grafting.

## Abbreviations

The following abbreviations are used in this manuscript:

BMI	Body mass index
CABG	Coronary artery bypass rafting
CCAB	Conventional on-pump coronary artery bypass
CI	Confidence interval
CPB	Cardiopulmonary bypass
ECMO	Extracorporeal membrane oxygenation
eGFR	Estimated glomerular filtration rate
HR	Hazard ratio
OPCAB	Off-pump coronary artery bypass
PBM	Patient blood management
RBC	Red blood cell
