# Supplementary material for: Perioperative Red Blood Cell Transfusion and Long-Term Mortality in Coronary Artery Bypass Grafting: On-Pump and Off-Pump Analysis
Source: J Clin Med. 2025 Apr 13;14(8):2662. doi: 10.3390/jcm14082662 (PMC12027956; doi:10.3390/jcm14082662)
Supplement: Supplementary file 1 [file jcm-14-02662-s001.zip › jcm-3527659-supplementary.pdf]

Supplementary Table S1. Multivariable analysis for overall death in conventional on pump coronary artery bypass grafting group

| **Variables** | **Hazard ratio (95% CI)** | ***P*-value** |
|---|---|---|
| eGFR (ml/min) | | |
| eGFR ≥90 | Reference | |
| eGFR <90 and ≥60 | 1.40(0.91-2.16) | 0.121 |
| eGFR <60 and ≥30 | 1.78(1.10-2.86) | 0.018 |
| eGFR <30 | 5.04(2.70-9.41) | <.0001 |
| Congestive heart failure | 1.65(1.23-2.22) | 0.001 |
| Malignancy history | 1.78(1.26-2.51) | 0.001 |
| Atrial fibrillation | 1.41(0.95-2.11) | 0.091 |
| Peripheral artery disease | 1.70(1.24-2.33) | 0.001 |
| Emergency admission | 1.36(1.01-1.84) | 0.045 |
| ECMO use | 11.61(8.13-16.58) | <.0001 |
| Surgery at high volume center | 0.62(0.45-0.87) | 0.005 |
| RBC transfusion | | |
| Number of RBC, 0 | Reference | |
| Number of RBC, 1 | 1.72(0.55-5.46) | 0.354 |
| Number of RBC, 2 ≤ | 2.96(0.97-9.06) | 0.057 |

* eGFR, estimated glomerular filtration rate; BMI, body mass index; ECMO, extracorporeal membrane oxygenation; RBC, red blood cell; OPCAB, off pump coronary artery bypass grafting; Surgery at high-volume centers (defined as centers performing more than 50% of surgery cases)

Supplementary Table S2. Multivariable analysis for overall death in off pump coronary artery bypass grafting

| Variables | Hazard ratio (95% CI) | *P*-value |
|---|---|---|
| Age (years) | | |
| 20-40 | Reference | |
| 40~60 | 0.79(0.33-1.89) | <.0001 |
| 60~80 | 1.52(0.66-3.50) | <.0001 |
| 80≤ | 2.82(1.17-6.80) | <.0001 |
| Female sex | 0.69(0.50-0.95) | 0.024 |
| eGFR (ml/min) | | |
| eGFR ≥90 | Reference | |
| eGFR <60 and ≥30 | 1.71(1.17-2.49) | 0.006 |
| eGFR <30 | 2.80(1.69-4.63) | <.0001 |
| Anemia | 1.21(0.76-1.92) | 0.429 |
| BMI (kg/m²) | | |
| < 18.5 | Reference | |
| 30 < | 0.31(0.09-1.07) | 0.064 |
| Congestive heart failure | 1.56(1.20-2.03) | 0.001 |
| Ischemic stroke | 1.53(1.16-2.02) | 0.003 |
| Malignancy history | 1.42(1.02-1.98) | 0.037 |
| Atrial fibrillation | 1.78(1.22-2.58) | 0.003 |
| Peripheral artery disease | 1.27(0.96-1.68) | 0.092 |
| Anti-platelet agent medication | 0.80(0.58-1.10) | 0.172 |
| ECMO use | 9.79(5.69-16.85) | <.0001 |
| Surgery at high volume center | 0.68(0.53-0.88) | 0.003 |
| RBC transfusion | | |
| Number of RBC, 0 | Reference | |
| Number of RBC, 1 | 1.34(0.90-2.00) | 0.152 |
| Number of RBC, 2 ≤ | 2.28(1.55-3.37) | <.0001 |

* eGFR, estimated glomerular filtration rate; BMI, body mass index; ECMO, extracorporeal membrane oxygenation; RBC, red blood cell; OPCAB, off pump coronary artery bypass grafting; Surgery at high-volume centers (defined as centers performing more than 50% of surgery cases)
